# Isabelle Olivieri (1957–2016)

**DOI:** 10.1111/eva.12453

**Published:** 2016-12-25

**Authors:** Louis Bernatchez

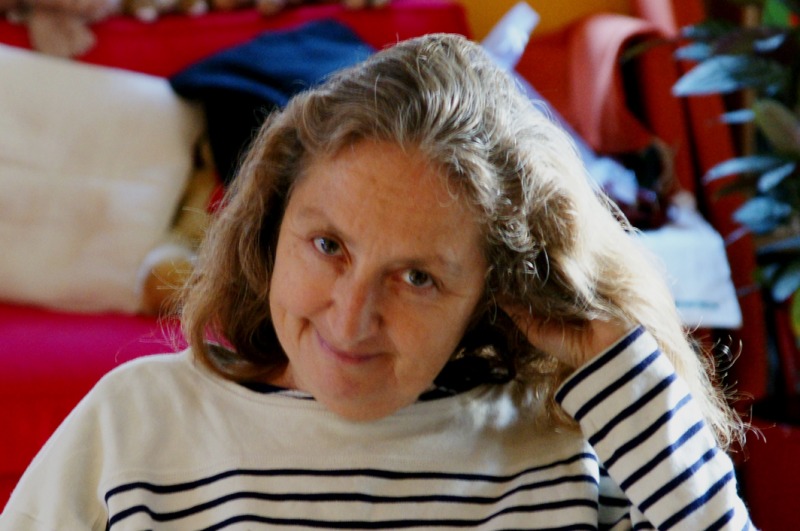

Isabelle Olivieri passed away on Saturday 10 December at 4 a.m. She passed away quietly after having fought not only with an admirable courage against her cancer but also against a paraneoplastic syndrome, which heavily handicapped her for the last years of her life. Isabelle would have passed 60 years in March 2017. She entered the Agronomic College (AgroParisTech) in Paris in 1977, specialized in zoology and then oriented her research towards evolutionary genetics. Her PhD work was concerned with Mediterranean thistles. These plants were invasive in Australia, and one of the goals of the CSIRO where she was based in Montpellier was to find control agents. She would come back to the question of plant–insect interactions later in her life. She did her postdoc in Paul Ehrlich's laboratory in Stanford in 1983 and there further developed her ideas on the importance of the metapopulation concept for the understanding of the evolution of migration mechanisms. The paper which she published on this subject in American Naturalist (after a more than 4 years debate with the editors) provided her with a congratulation letter from Ernst Mayr (this letter is probably somewhere in her “records,” Isabelle has never been very gifted for ordering, her papers as the rest!).

Hired at the INRA (Agronomic Research Institute) in a laboratory near Montpellier on a programme concerning Mediterranean alfalfa, predominantly annuals, she developed her research on the evolution of genetic systems by studying the evolutionary mechanisms determining the lifespan of organisms according to their ecology. Her DSc thesis will treat of the constitution of an integrative approach of evolution uniting demographic and genetic models on both migration and longevity characters.

Hired as full professor at Montpellier, she became the first professor in Population Genetics at Montpellier and set up high‐level courses. She carried on with her quest of evolutionary mechanisms by coming back to thistles and their insects. She studied habitat choice, host specificity, realized a leading study on the conservation biology of a local *Centaurea*, again in relation to the metapopulation concept, and developed experimental evolution studies. The list of questions which she tackled with her team or with diverse collaborators would be too long to be detailed here.

Isabelle also became a recognized actor of international evolutionary biology. John Maynard Smith has once said that she was the fifth column^1^ of the French evolutionary biology into the Anglo‐Saxon world. Indeed, she became the president of the European Society of Evolutionary Biology and the vice president of the American Society for the Study of Evolution, the Dutch Academy of sciences attributed her a comfortable grant allowing her to come whenever and wherever she wishes in any university to interact with local scientists, she was a member of the editorial board of numerous journals, and the very prestigious EMBO (European Molecular Biology Organization) elected her as a member precisely for her ability to open new fields. Also, the French Society of Ecology gave her its “grand prix” and CNRS its silver medal. Despite all those recognition proofs, Isabelle did not search for honours nor for responsibilities. Actually, she would rather make her best to escape from them. She, however, used all opportunities to defend her convictions and transmit messages. Invited by the French president to a lunch with other female scientists, she gave an interview to a newspaper criticizing his policy for scientific research. In her national and international activities, Isabelle has established sustainable and fruitful cooperative relationships and tried to gather ideas and people together and bring demography and genetics, ecology and evolution together. It was undoubtedly one of the causes of her charisma, of the fact that a number of us were ready to follow her even on steep paths, just like the Corsican ones she liked so much.

When she passed her DSc, Georges Valdeyron, who had supervised her PhD and who was 45 older than her, stated that the students he was interacting with were a bit like his progeny but that his relation with her was different; actually, she had been more like a nursing mother. From that point of view, in a way, we, French evolutionary biologists, are orphans today. The one who dared say things which should not be told, who disputed fiercely, step by step—let us admit it, it was sometimes painful—any opinion which she did not find rational, the one who oriented the research of a lot of us and who was an example for many has disappeared. If Isabelle could be severe, brusque sometimes, all of those who have known her have benefited from her exceptional generosity and from her care for collective actions. Her involvement at the collective level has led those who worked with her to establish deep affective relations with her and to build this large family, of which she is the soul, and which remains united now that she has gone. Her house she shared with her companion was widely open, and innumerable people have spent unforgettable moments there. We send our friendly thoughts to her family; they can be assured that her memory and her works will remain, lasting, in our community.

Le 10 décembre 2016

Pierre‐Henri Gouyon, Agnès Mignot, Ophélie Ronce, Myriam Valéro

*Editor's note:*

Besides all of her outstanding academic accomplishments, Isabelle has been involved with Evolutionary Applications as an associate editor since the onset of the journal nearly 10 years ago. Isabelle enjoyed broad knowledge in evolutionary biology meaning that I could send her any type of paper knowing that she would handle them efficiently and thoroughly every time. Despite the serious health issues that affected her, she remained active and involved until the end. In fact, I handled the last paper that she was responsible for only 4 days before she passed away. The last scientific paper that Isabelle wrote was with her colleagues Jeanne Tonnabel, Ophélie Ronce and Agnes Mignot. The paper was called “Why evolution matters for species conservation: perspectives from three case studies of plant metapopulations” and appeared in the 2016 Special Issue of Evolutionary Applications “Women's contribution to basic and applied evolutionary biology.”

We will all miss her a lot. Merci pour tout Isabelle…

## Note

1 i.e. any group of people who undermine a larger group.

